# Anomalies électrocardiographiques et échocardiographiques au cours de la cirrhose virale b: à propos de 60 cas au service d’hepato-gastroenterologie de l’Hopital Aristide Le Dantec de Dakar (HALD)

**DOI:** 10.11604/pamj.2018.30.169.12344

**Published:** 2018-06-25

**Authors:** Mamadou Ngoné Guèye, Bassène Marie Louise, Bodian Malick, Diallo Salamata, Thioubou Mame Aïssé, Halim Ambdil, Fall Marième Polèle, Cissé Cheikh Ahmadou Bamba, Dia Daouda, Mbengue Mouhamadou, Ba Serigne Abdou, Diouf Mamadou Lamine

**Affiliations:** 1 Service d’Hépato, Gastroentérologie HALD, Senegal; 2 Service d’Hépato-Gastroentérologie Hôpital Général de Grand Yoff, Senegal; 3 Service de Cardiologie HALD, Senegal

**Keywords:** Cirrhose, VHB, cardiomyopathie, Keywords, Cirrhosis, HBV, cardiomyopathy

## Abstract

L’objectif de cette étude est d’évaluer les anomalies électrocardiographiques et échocardiographiques chez les patients atteints de cirrhose virale B et identifier leurs déterminants. Il s’agissait d’une étude prospective sur 8 mois aux services de d’Hépato-Gastroentérologie et de Cardiologie de l’hôpital Aristide Le Dantec de Dakar. Tous les patients atteints de cirrhose B virale et n’ayant pas de cardiopathie pré existante connue ont été inclus. Nous avons collecté et analysé les données épidémiologiques, cliniques, biologiques, échographiques, endoscopiques, électrocardiographiques et échocardiographiques (2D et Doppler) de tous les patients. Les données ont été saisies sur tableau Excel 2007 et analysées à l’aide du logiciel Sphinx version 5. La comparaison des variables a été faite par les tests de Chi 2 standard et Fisher. Le seuil de significativité a été arrêté à p< 0,05 pour ces 2 tests statistiques. Soixante patients ont été inclus. La prévalence de la cirrhose virale B était de 3,4%. L’âge moyen était de 41 ans et le sex ratio de 1,6 (37 hommes). La cirrhose était classée Child-Pugh B chez 29 patients (49%), Child-Pugh C et Child Pugh A respectivement chez 20 patients (33%) et 11 patients (18%). Les anomalies électrocardiographiques les plus fréquentes étaient l’hypertrophie ventriculaire gauche et l’allongement de l’intervalle QTc objectivés respectivement chez 27 patients (45%) et 24 patients (40%). L’analyse statistique a montré une association entre l’intervalle QTc prolongé et la gravité de la cirrhose (p = 0,01, RR = 2, IC = 0,24 à 0,341). Les anomalies échocardiographiques étaient dominées par la dilatation ventriculaire gauche (58,3%) et l’hyperdébit cardiaque (43,3%) avec un débit moyen de 6,05 l/min. L’analyse stastistique mettait en évidence une association significative entre la gravité de la cirrhose et l’hyperdébit cardiaque (p = 0,003; IC: 95% (de 2,883 à 38,58; RR = 2). Au total, 14 patients (23,3%) avaient une cardiomyopathie cirrhotique latente. La cirrhose virale B peut induire des anomalies cardiaques diverses et variées. Ces anomalies sont morphologiques et/ou électrophysiologiques et leur gravité semble corrélée à la sévérité de la cirrhose.

## Abstract (en)

This study aimed to evaluate the electrocardiographic abnormalities in patients with cirrhosis due to viral hepatitis B and to identify their determinants. We conducted a prospective study in the Hepatogastroenterologic Department and in the Department of Cardiology at the Aristide Le Dantec Hospital in Dakar over a period of 8 months. All patients with cirrhosis due to viral hepatitis B and without a history of heart disease were included in the study. We collected and analyzed the epidemiological, clinical, echographic, endoscopic, electrocardiographic, echocardiographic (2D and Doppler) data and laboratory data from all patients. Sixty patients were enrolled. The prevalence of cirrhosis due to viral hepatitis B was 3.4%. The average age was 41 years and the sex-ratio was 1.6 (37 men). Cirrhosis was classified as Child-Pugh B in 29 patients (49%), Child-Pugh C and Child Pugh A in 20 patients (33%) and 11 patients (18%) respectively. The most common electrocardiographic abnormalities included left ventricular hypertrophy and QTc interval prolongation detected in 27 patients (45%) and 24 patients (40%) respectively. Statistical analysis showed an association between prolonged QTc interval and the severity of cirrhosis (p = 0.01, RR = 2, CI = 0.24 - 0.341). Echocardiographic abnormalities were dominated by left ventricle dilatation (58.3%) and cardiac hyperoutput (43.3%), with an average output of 6.05 l/ min. Statistical analysis revealed a significant association between cirrhosis severity and cardiac hyperoutput (p = 0.003; CI: 95%, 2,883-38,58; RR = 2). A total of 14 patients (23.3%) had latent cirrhotic cardiomyopathy. Cirrhosis due to viral hepatitis B can cause a wide range of different cardiac abnormalities. These include morphological and/or electrophysiological abnormalities whose severity appears to be correlated with cirrhosis severity.

## Introduction

La cirrhose, stade ultime de plusieurs maladies chroniques du foie peut se compliquer d'une atteinte cardiaque spécifique nommée cardiomyopathie cirrhotique. Décrite par Lee en 1989, la cardiomyopathie cirrhotique correspond à une constellation d'anomalies cardiaques structurelles et fonctionnelles relevées chez le cirrhotique, en dehors de toute pathologie cardiaque sous-jacente [1, 2]. En Occident, depuis la fin des années 80, de nombreuses études se sont intéressées à l'atteinte cardiaque au cours de la cirrhose, notamment celle d'origine alcoolique. En Afrique subsaharienne particulièrement au Sénégal, où l'infection chronique par le VHB représente la première cause de cirrhose, aucune étude à notre connaissance n'a évalué l'état du c'ur au cours de la cirrhose virale B. Les objectifs de cette étude étaient d'une part de décrire les anomalies électrocardiographiques et échocardiographiques des patients atteints de cirrhose virale B et d'identifier leurs déterminants, d'autre part de préciser la fréquence de la cardiomyopathie cirrhotique.

## Méthodes

Il s'agissait d'une étude transversale, descriptive et analytique couvrant la période allant du 1er Avril 2014 au 30 Novembre 2014 menée aux services d'hépato-gastroentérologie et de cardiologie de l'Hôpital Aristide Le Dantec de Dakar (HALD). La population d'étude était constituée par l'ensemble des patients reçus en consultation ou hospitalisés dans le service d'hépato-gastroentérologie de l'HALD quelle que fût le motif. Étaient inclus tous les patients ayant une cirrhose virale B. Le diagnostic positif de la cirrhose virale B reposait sur un faisceau d'arguments cliniques, biologiques, échographiques, endoscopiques et immunologiques et celui de la cardiomyopathie cirrhotique sur une fonction systolique altérée au repos (fraction d'éjection systolique < 55%) ou une dysfonction diastolique ventriculaire (rapport E/A < 1 associé à un temps de décélération > 200 ms pour les patients de moins de 50 ans et > 240 ms pour les plus de 50 ans), avec ou sans allongement de l'intervalle QTc. Les critères de non inclusion étaient: une cardiopathie préexistante connue ou une cause connue de dysfonction myocardique (maladie coronarienne, diabète sucré, hypertension artérielle, anémie sévère), une notion d'alcoolisme chronique, une co-infection par le VHC, le VHD ou le VIH, une cirrhose dégénérée. Les données ont été saisies sur tableau Excel 2007 et analysées à l'aide du logiciel Sphinx version 5. La comparaison des variables a été faite par les tests de Chi 2 standard et Fisher. Le seuil de significativité a été arrêté à p < 0,05 pour ces 2 tests statistiques.

## Résultats

**Figure 1:**
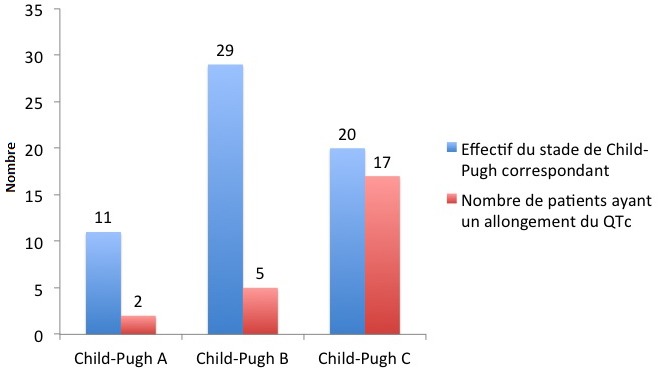
Répartition des patients présentant un allongement de l’intervalle QTc en fonction de leur score Child-Pugh

Au cours de la période d'étude, 1740 patients ont été reçus en consultation, ou admis en hospitalisation au service d'hépato-gastroentérologie de l'hôpital Aristide Le Dantec. Nous avons inclus 60 patients. La prévalence de la cirrhose virale B était de 3,4%. Le sex-ratio était de 1,6 (37 hommes). L'âge moyen était de 41 ans avec des extrêmes de 18 et 72 ans. Les principales manifestations cliniques étaient la splénomégalie (60%), l'ascite (58,3%), l'ictère cholestatique (43,3%) et l'hépatomégalie d'allure cirrhotique (28,3%). L'hémogramme objectivait une anémie normochrome normocytaire dans 45% des cas, une thrombopénie et une leucopénie respectivement dans 60% et 48,3% des cas. Les explorations fonctionnelles hépatiques montraient une cholestase dans 55% des cas, une cytolyse dans 60% avec une prédominance sur les ASAT dans 94,4% et une insuffisance hépatocellulaire dans 80%. L'urée sanguine, la créatinémie et la calcémie corrigée étaient normales chez tous les patients. La cirrhose était classée Child-Pugh B chez 29 patients (49%), Child-Pugh C et Child Pugh A chez respectivement 20 (33%) et 11 patients (18%). L'ADN viral B a été quantifié dans 43,3% des cas. Il était détectable dans 46,2% des cas et son taux variait entre 122 UI/ml et 23 225 10 3 UI/ml. La charge virale moyenne était de 1235 UI/ml. Les anomalies objectivées à l'électrocardiogramme étaient multiples (Tableau 1). Les plus fréquentes étaient l'hypertrophie ventriculaire gauche décelée chez 27 patients (45%), l'allongement de l'intervalle QTc et la tachycardie sinusale objectivée respectivement dans 24 cas (40%) et 17 cas (28,3%). Dix sept des 24 patients qui présentaient un allongement de l'intervalle QTc étaient au stade C de Child-Pugh (Figure 1). L'analyse statistique a mis en évidence une association entre l'allongement de l'intervalle QTc et la sévérité de la cirrhose (p = 0,01, RR = 2; IC = 0,24-0,341). L'échocardiographie, de même mettait en évidence de multiples anomalies (Tableau 2). Elles étaient dominées par la dilatation du ventricule gauche (58,3%), l'hyperdébit cardiaque (43,3%) avec un débit moyen de 6,05 l /min et des extrêmes de 3,7 l/min et 10,8 l/min et la dysfonction diastolique du ventricule gauche (20%). Parmi les patients qui présentaient un hyperdébit cardiaque, 12 (38,5%) avaient une anémie. Onze patients parmi les 16 qui avaient hyperdébit cardiaque sans anémie, étaient au stade C de Child pugh. Une association entre la gravité de la cirrhose et l'hyperdébit cardiaque était objectivée chez les patients n'ayant pas d'anémie: p = 0,003; IC: 95% (2,883-38,58); RR = 2. Au total, 14 patients (23,3%) présentaient une cardiomyopathie cirrhotique latente.

**Tableau 1:** Anomalies ECG observées et leur association avec la sévérité de la cirrhose

Anomalies ECG	Effectifs	Pourcentage	P= value
Arythmie sinusale	5	8,3	p=0,8
Tachycardie	17	28,3	p=0,2
Bradycardie	4	6,7	p=0,4
BAV 1èr degré	2	3,3	p=0,6
Bloc de branche droit	2	3,3	p=0,6
HAG	21	35	p=0,1
HAD	7	11,6	p=0,7
HVG	27	45	p=0,1
HVD	1	1,7	p=0,8
Allongement QTc	24	40	p=0,01

HAG= hypertrophie auriculaire gauche ; HAD= hypertrophie auriculaire droite

HVG= hypertrophie ventriculaire gauche ; HVD= Hypertrophie ventriculaire droite

BAV= Bloc auriculo-ventriculaire ; QTc= QT corrigé

**Tableau 2:** Anomalies ECG observées et leur association avec la sévérité de la cirrhose

Anomalies échocardiographiques	Effectifs	Pourcentages	P=value
Hyperdébit cardiaque sans anémie	16	26 ,7	P=0,001
Baisse fonction systolique VG	2	3,3	P=0,34
Dysfonction diastolique VG	12	20	P=0,1
Dilatation ventricule gauche	35	58,3	P=0,08
Dilatation oreillette gauche	18	30	P=0,46
Epaississement SIV	16	26,6	P=0,23

SIV= Septum inter ventriculaire ; VG= Ventricule gauche

## Discussion

Les principales anomalies électrocardiographiques observées dans notre étude étaient l'allongement du QTc, objectivé dans 40% des cas ainsi que l'HVG et l'HAG présentes respectivement dans 45% et 35%. Ces résultats étaient superposables aux données de la littérature. Tint et al en Roumanie [3] rapportaient dans une série de 57 patients cirrhotiques un allongement du QTc dans 33%. Au Danemark, Møller S et al rapportaient un allongement du QTc, une HVG et une HAG dans respectivement 45%, 58% et 38% des cas [4]. L'allongement de l'intervalle QTc est objectivé chez environ 30 à 60% des patients cirrhotiques selon les séries [2]. Il résulte d'une repolarisation anormale du myocarde.Une association statistiquement significative entre l'allongement de l'intervalle QTc et la sévérité de la cirrhose virale B était observée au cours de notre étude. Cette même association était mise en évidence dans plusieurs études [3, 5]. Une tachycardie sinusale était objectivée dans 28,3% des cas. Tint et al et Hansen et al rapportaient des prévalences plus élevées qui étaient respectivement de 38,6% et 44% [3, 6]. Cette différence serait liée à la proportion importante de patients sous béta bloquants dans notre série. En effet, du fait du diagnostic tardif de la cirrhose dans nos régions, la majeure partie des patients présentent des signes endoscopiques d'HTP au moment du diagnostic et sont sous béta bloquants. Ainsi, 88,3% de nos patients avaient un traitement par béta bloquants contre respectivement 71% et 62% dans les séries roumaines et danoises [3, 6]. Les anomalies morphologiques étaient dominées par la dilatation du ventricule gauche, objectivée dans 58,3% des cas, suivie de la dilatation de l'oreillette gauche et l'hypertrophie du septum interventriculaire présents respectivement dans 30% et 26,6% des cas. Il n'existait pas d'association statistiquement significative entre la sévérité de la cirrhose et ces anomalies morphologiques. Tint D et al rapportaient une dilatation du ventricule gauche dans 70% des cas et une dilatation de l'OG dans 44% des cas [3]. Ces taux élevés de dilatations des cavités cardiaques gauches pourraient en partie être liés à l'anémie chronique fréquemment rencontrée au cours de la cirrhose. En effet, l'anémie chronique entraine une grande variété de changements adaptatifs dans le système cardiovasculaire dont, notamment une dilatation ventriculaire. Dans notre étude, 34,8% des patients présentant une dilatation du ventricule avaient une anémie. L'hyperdébit cardiaque était objectivé dans 43,3% des cas. Ce résultat était comparable à ceux rapportés par Cohen-Solal et al et Moller S et al qui trouvaient respectivement un hyperdébit cardiaque au cours de la cirrhose alcoolique dans 48% et 44% [7, 8]. Cet hyperdébit cardiaque observé est indépendant de l'étiologie de la cirrhose et semble être fonction de la sévérité de celle-ci.En effet, il existait une association statistiquement significative entre l'hyperdébit cardiaque et la sévérité de la cirrhose virale B chez les patients n'ayant pas d'anémie. Cette association est décrite dans la littérature. Toutefois, chez les patients qui présentaient une anémie, l'hyperdébit cardiaque pourrait être lié à celle-ci, d'où l'intérêt de contrôler cet hyperdébit après correction de l'anémie. La FEVG n'était abaissée que dans 3,3% des cas. Ce résultat était nettement inférieur à ceux de Mandell MS et al. et de Wong F et al qui rapportaient une baisse de la FEVG dans respectivement 36,7% et 43,6% [9, 10]. Cette différence s'explique par le fait qu'à l'état basal, la fraction d'éjection du ventricule gauche est normale ou supra normale. Contrairement à notre étude où il s'agissait exclusivement d'échocardiographie de repos, Mandell MS et Wong F ont réalisé des échographies de stress, expliquant ainsi cette prévalence relativement élevée d'altération de la FEVG. L'échographie de stress ou à la dobutamine, souvent réalisée pour déceler une ischémie myocardique, peut se révéler intéressante afin de débusquer une baisse de la FEVG. En effet, l'étude de Kim MY et al [11]. a démontré l'intérêt de cet examen pour le dépistage de la dysfonction systolique au cours de la cardiomyopathie cirrhotique.

## Conclusion

La cirrhose virale B peut induire des anomalies cardiaques diverses et variées. Ces anomalies sont morphologiques et/ou électrophysiologiques et leur gravité semble corrélée à la sévérité de la cirrhose. Concept relativement nouveau et longtemps négligé, la cardiomyopathie cirrhotique constitue un élément qu'il faudra s'atteler à dépister, compte tenu des implications pronostiques.

### Etat des connaissances actuelles sur le sujet

- En Afrique subsaharienne où l'infection chronique par le VHB représente la première cause de cirrhose, aucune étude à notre connaissance n'a évalué l'état du c'ur au cours de la cirrhose virale B;
- En occident,des anomalies cardiaques ont été décrites au cours de la cirrhose alcoolique.

### Contribution de notre étude à la connaissance

- La cirrhose virale B peut induire des anomalies cardiaques diverses et variées;
- Ces anomalies sont morphologiques et/ou électrophysiologiques et leur gravité est corrélée à la sévérité de la cirrhose.

## Conflits d’intérêts

Les auteurs ne déclarent aucun conflit d'intérêts.
